# Cell surface protease activation during RAS transformation: Critical role of the plasminogen receptor, S100A10

**DOI:** 10.18632/oncotarget.10279

**Published:** 2016-06-24

**Authors:** Patricia A. Madureira, Alamelu G. Bharadwaj, Moamen Bydoun, Katy Garant, Paul O'Connell, Patrick Lee, David M. Waisman

**Affiliations:** ^1^ Centre for Biomedical Research (CBMR), University of Algarve, Campus of Gambelas, Faro, Portugal; ^2^ Department of Biochemistry and Molecular Biology, Dalhousie University, Halifax, Nova Scotia, Canada; ^3^ Department of Pathology, Dalhousie University, Halifax, Nova Scotia, Canada

**Keywords:** S100A10, RAS, plasminogen, plasmin, annexin A2

## Abstract

The link between oncogenic *RAS* expression and the acquisition of the invasive phenotype has been attributed to alterations in cellular activities that control degradation of the extracellular matrix. Oncogenic RAS-mediated upregulation of matrix metalloproteinase 2 (MMP-2), MMP-9 and urokinase-type plasminogen activator (uPA) is critical for invasion through the basement membrane and extracellular matrix. The uPA converts cell surface-bound plasminogen to plasmin, a process that is regulated by the binding of plasminogen to specific receptors on the cell surface, however, the identity of the plasminogen receptors that function in this capacity is unclear. We have observed that transformation of cancer cells with oncogenic forms of *RAS* increases plasmin proteolytic activity by 2- to 4-fold concomitant with a 3-fold increase in cell invasion. Plasminogen receptor profiling revealed RAS-dependent increases in both S100A10 and cytokeratin 8. Oncogenic *RAS* expression increased *S100A10* gene expression which resulted in an increase in S100A10 protein levels. Analysis with the RAS effector-loop mutants that interact specifically with Raf, Ral GDS pathways highlighted the importance of the RalGDS pathways in the regulation of S100A10 gene expression. Depletion of S100A10 from RAS-transformed cells resulted in a loss of both cellular plasmin generation and invasiveness. These results strongly suggest that increases in cell surface levels of S100A10, by oncogenic RAS, plays a critical role in RAS-stimulated plasmin generation, and subsequently, in the invasiveness of oncogenic RAS expressing cancer cells.

## INTRODUCTION

It is now well established that a key requirement for invasion and metastasis of cancer cells is the degradation of both the extracellular matrix (ECM) that surrounds the tumor and the basement membrane which presents the final barrier between the tumor cells and the blood stream [1–5]. Degradation of the ECM facilitates invasion of tumor cells into the organ parenchyma, whereas degradation of the basement membrane is required for their entry into the blood stream as well as their metastasis to distant organs. Cancer cells that have acquired an invasive phenotype release proteases that attack and digest the protein component of the matrix that would restrict the movement of these malignant cells. These proteases, including the urokinase-type plasminogen activator (uPA) [6, 7], cathepsin B [8] and matrix metalloproteinases (MMPs) [9], have been the subject of intense study in recent years as potential mediators of cancer cell invasion and metastasis. For example, multiple studies have demonstrated that the expression of uPA is directly linked to tumor cell invasiveness and metastasis [10–13]. Importantly, uPA expression in both the tumor and the surrounding stroma has been shown to be a prognostic indicator of survival in various cancers with high expression being associated with poor prognosis [5, 14–17].

The uPA is secreted from cells as an inactive proenzyme, pro-uPA, which binds to the cell surface via its cell surface receptor, uPAR. The latter is associated with the external surface of the plasma membrane by a glycosyl phosphatidylinositol (GPI) anchor, and functions to localize pro-uPA to the cell surface where it is activated to form the active serine protease, uPA. The uPA cleaves plasminogen, generating the active protease plasmin. Plasmin is a broad specificity serine protease. It degrades fibrin in blood clots (fibrinolysis) and other substrates, including components of the extracellular matrix and basement membranes such as type IV collagen, fibronectin and laminin [18–20]. Plasmin also activates MMPs including pro-MMP-1, −3, −7, −9, −10, and −13, resulting in further breakdown of the ECM [21, 22]. The plasminogen that serves as a substrate for uPA is localized to the cell surface due to its interaction with specific plasminogen receptors [23–28].

S100A10 is a key plasminogen receptor that binds to the cell surface via its cell surface receptor, annexin A2 [29, 30]. The S100A10/annexin A2 complex colocalizes with the uPA/uPAR complex thereby localizing plasmin generation to the cell surface [31, 32]. S100A10 regulates between 50–90% of the plasmin generation of a number of normal and cancer cells (reviewed in [24, 33]). The first link between plasminogen receptors and oncogenic transformation of cells was the report that S100A10 was upregulated by the oncogene responsible for acute promyelocytic leukemia (APL), PML-RARα [34]. These studies showed that expression of PML-RARα resulted in enhanced plasmin activity and increased cell surface expression of S100A10. When PML-RARα expressing cells were depleted of S100A10 and expression of PML-RARα initiated, plasmin generation was dramatically decreased. Thus, S100A10 was shown to be directly regulated by and to play a critical role in the stimulation of plasmin generation by the PML-RARα oncoprotein.

The acquisition of activating mutations in the *RAS* gene family results in the progression of precancerous cells to malignancy. The expression of the oncogenic RAS protein, one of the earliest oncogenic events in many cancers, also increases the expression of pro-uPA and uPAR [35, 36]. This RAS-dependent activation of uPA/uPAR is thought to account, in part, for increases in cellular proteolytic activity, although a link between RAS- dependent transformation and increased cellular plasmin proteolytic activity has not been directly demonstrated. In the current report, we have investigated the regulation of plasminogen receptors by oncogenic RAS and their relationship to RAS-dependent changes in plasmin generation and cellular invasion. This study identifies for the first time, the plasminogen receptor, S100A10, as a key link between RAS-dependent oncogenic transformation of cells and RAS-dependent increases in plasmin proteolytic activity and cancer cell invasion.

## RESULTS

### Expression of oncogenic RAS stimulates cellular plasmin generation

**Figure 1:**
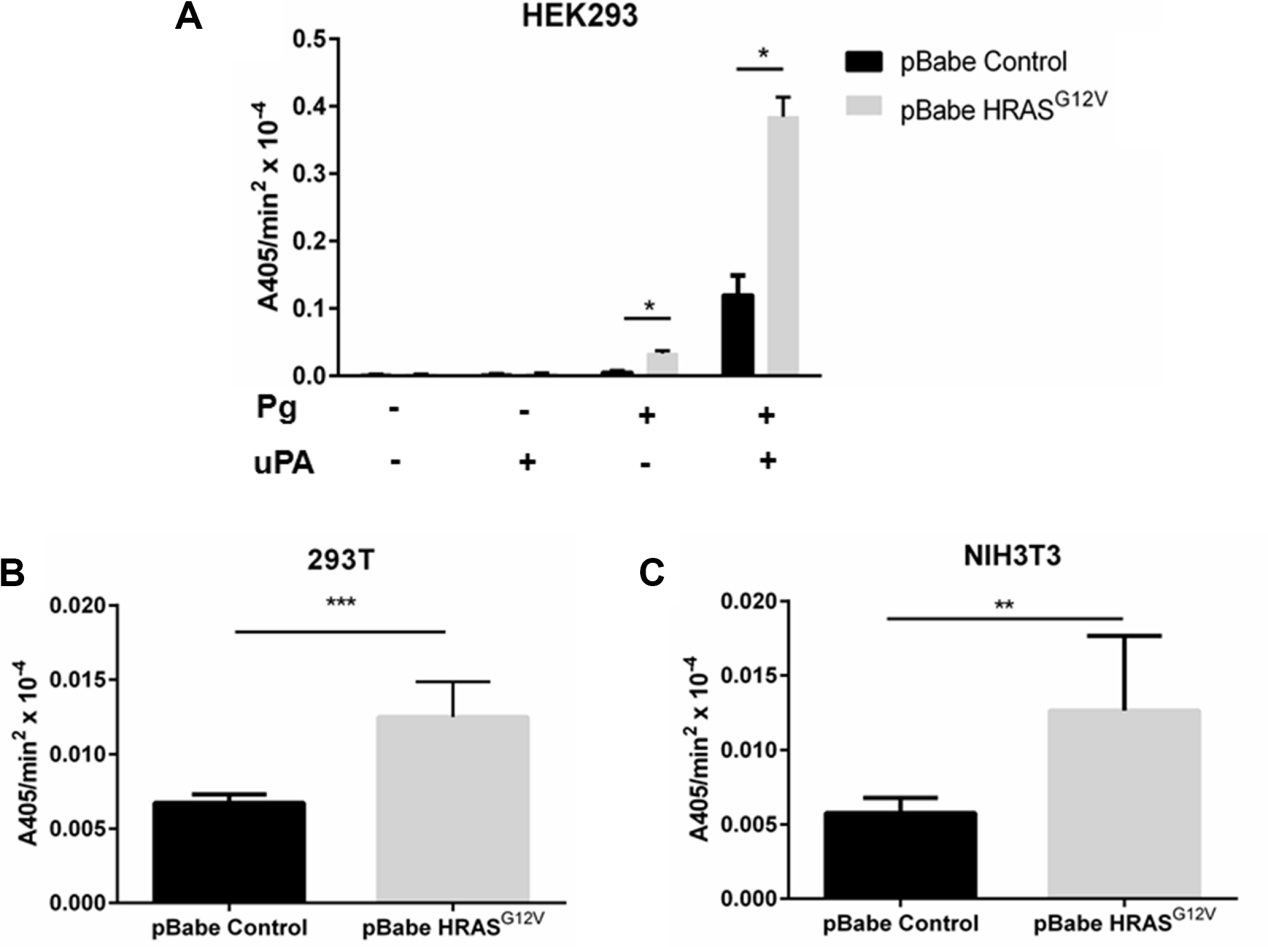
The expression of oncogenic Ras activates cellular plasmin generation HEK 293 (**A**), 293T (**B**), NIH-3T3 (**C**) were transduced with either empty vector retrovirus (pBabe control), or oncogenic HRAS G12V expressing retrovirus (HRAS^G12V^) and incubated with 1 μM glu-plasminogen and 50 nM uPA for 10 minutes before the addition of 500 μM plasmin substrate S2251. The rate of plasmin generation was determined from the slope of the A405 nm vs time2 progress curve (*N* = 6). Statistical analysis was performed by Student's *t*-test.

The link between oncogenic RAS expression and the acquisition of the invasive phenotype has been attributed to alterations in cellular activities that regulate the degradation of the extracellular matrix (reviewed in [37]). Although the RAS-dependent regulation of the MMPs and cathepsin B has been well established [37–39], it has not been clear to what extent plasmin activity is regulated by oncogenic RAS. In order to determine if *RAS* transformation affects cellular plasmin generation, we transfected HEK 293 cells with an empty vector (HEK-293-pBABE control) or with the oncogenic *HRAS* (G12V) mutant (HEK-293-HRAS) and measured plasmin generation. Since expression of oncogenic RAS can increase the release of the plasminogen activator, urokinase-type plasminogen activator (uPA), cells were assayed both in the presence and absence of exogenous uPA. As shown in Figure 1A, expression of oncogenic HRAS results in a three-fold increase in plasmin proteolytic activity in the presence of exogenous uPA and a five-fold increase in plasmin proteolytic activity in the absence of exogenous uPA. We also observed that expression of oncogenic HRAS increased plasmin proteolytic activity by about 2-fold in 293T and NIH-3T3 cell lines (Figure 1B, 1C). Furthermore, the expression of wild-type HRAS or oncogenic KRAS also increased plasmin proteolytic activity (Supplementary Figure S1). A RAS-GTP pulldown assay and subsequent western blot analysis confirmed increased RAS activity in RAS-transfected cell lines (Supplementary Figure S2). These data establish that expression of different members of the RAS family increases cellular plasmin generation in several cell lines.

### Oncogenic RAS-dependent activation of plasminogen is mediated by a plasminogen receptor with a carboxyl-terminal lysine

**Figure 2:**
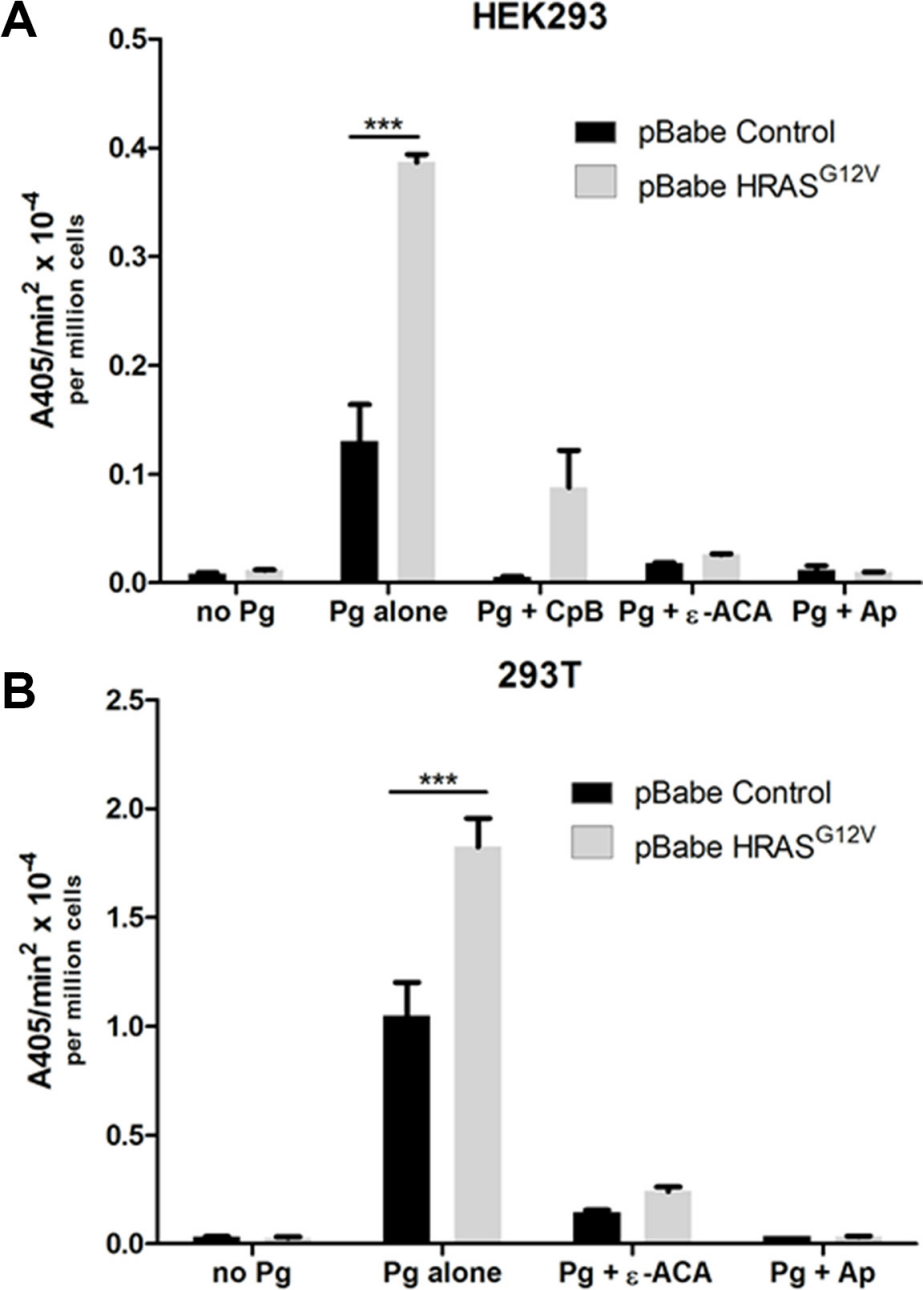
Mechanism of plasmin generation at the cell surface of oncogenic RAS expressing cells Plasmin generation was measured for (**A**), HEK 293 or (**B**), 293T cells transduced with either empty vector retrovirus (pBabe control) or oncogenic HRAS G12V expressing retrovirus (HRAS^G12V^) and stably selected with puromycin. Cells were either plated in 96 well plates for two days (HEK 293) before assay or released with enzyme free cell dissociation buffer and plated in 96 well plates in suspension for the assay (293T). Cells were either mock treated or treated with aprotinin (Ap) (2.2 μM), ε-ACA (100 mM) or carboxypeptidase B (CpB) (5U/ml) as indicated. The cells were washed 3 times with incubation buffer (Hanks balanced salt solution containing 3 mM CaCl_2_ and 1 mM MgC_l2_) and incubated with 0.5 μM glu-plasminogen for 20–30 minutes with or without aprotinin and ε-ACA before the addition of 500 μM plasmin substrate S2251. For the CpB treatments, cells were incubated with or without CpB for 30 minutes and washed three times before the addition of 0.5 μM glu-plasminogen. The rate of plasmin generation was determined from the slope of the A405 nm vs min^2^ progress curve (*N* = 4). Statistical analysis was performed by two-way ANOVA.

Plasmin generation results from the interaction of plasminogen activators with cell surface bound plasminogen. Although a single receptor has been extensively characterized for uPA, namely uPAR, multiple plasminogen receptors have been identified on the surface of normal and transformed cells [25, 26, 40, 41]. Mechanistically, the binding of plasminogen to its cell surface receptors involves the interaction of the plasminogen kringle domains with lysine residues of plasminogen receptors [27, 28, 42, 43]. It is generally accepted that plasminogen receptors with carboxyl- terminal lysine residues are most effective in both plasminogen binding and subsequent plasmin generation although internal lysines have also been shown to interact with plasminogen [44, 45]. We observed that pretreatment of cells with the lysine mimetic, ε-aminocaproic acid (ε-ACA), which binds to and blocks the interaction of the plasminogen kringle domains with plasminogen receptors, inhibited plasmin generation by control and H-RAS transformed HEK-293 (Figure 2A) and 293T cells (Figure 2B) by better than 80%. This established the importance of the lysine-binding regions of the plasminogen kringle domains in plasmin generation. We also observed that removal of the extracellular carboxyl- terminal lysine residues from cell surface receptor proteins, by pretreatment of cells with carboxypeptidase B, resulted in a significant reduction in plasmin generation by HEK-293 cells (Figure 2A), thus highlighting the importance of plasminogen receptors with carboxyl-terminal lysines in the RAS-stimulated generation of plasmin. Pretreatment of cells with the plasmin inhibitor, aprotinin resulted in a similar reduction in plasmin generation as carboxypeptidase B treatment. Furthermore, regardless of whether plasmin activity was stimulated by oncogenic HRAS, KRAS or wild-type HRAS, RAS- dependent plasmin generation was blocked by ε-ACA further confirming a key role for the lysine residues of the plasminogen receptor(s) in RAS-dependent plasmin generation (Figure 2 and Supplementary Figure S1).

### Plasmin plays a key role in RAS-dependent cellular invasiveness

**Figure 3:**
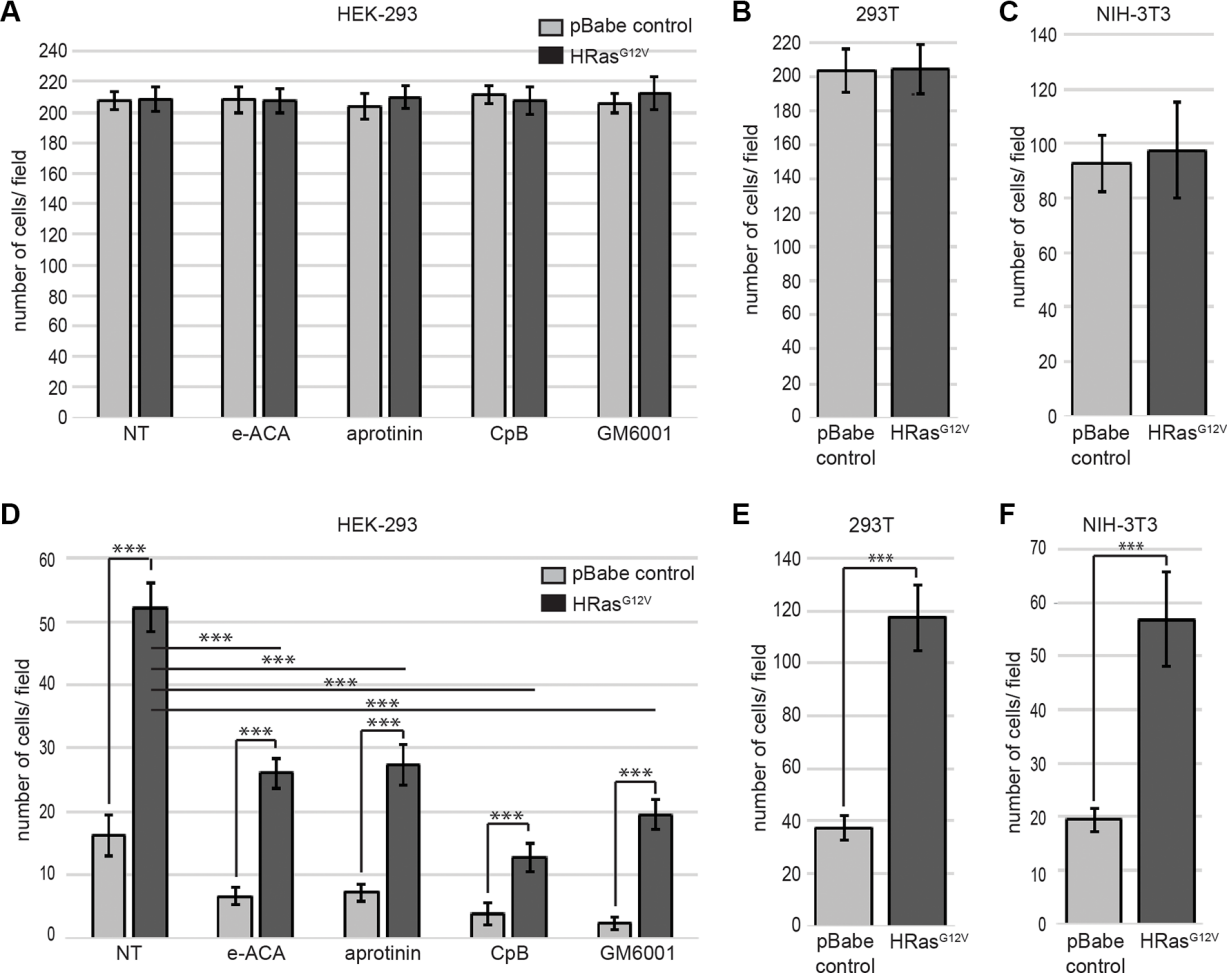
Enhanced plasmin generation plays a major role in oncogenic RAS-dependent cell invasion HEK 293 (**A**, **D**), 293T (**B**, **E**), or NIH-3T3 (**C**, **F**) cells were transduced with either empty vector retrovirus (pBabe control) or oncogenic HRAS G12V expressing retrovirus (HRAS^G12V^). Cells were plated on the upper chamber of uncoated chambers (A–C) or Matrigel (MTG)-coated chambers (**D**–**F**) and incubated in serum free medium containing 0.5 μM plasminogen, in the presence or absence of aprotinin (10 μM), ε-ACA (100 mM), CpB (5 U/ml) or GM6001 (10 μM), as indicated. The lower chamber contained medium with fetal bovine serum (FBS) as a chemoattractant. Invading cells were quantified according to the manufacturer›s instructions. Data are expressed as mean number of cells per 40× field (10 fields/experimental condition) plus or minus SD of 4 independent wells. Statistical analysis was performed by Student's *t*-test.

The initial step in the metastatic cascade is the activation of local tumor cell invasion, a process that has been termed invasive escape and that relies on the ability of cancer cells to break away from the primary tumor [11, 12]. The link between oncogenic RAS expression and the acquisition of the invasive phenotype has been attributed to the increased expression and/or activity of various proteases, including plasmin. Although the induction of uPA expression by oncogenic RAS has been well established, the direct role that oncogenic RAS plays in plasmin generation has not been studied in detail. Interestingly, we observed that although HRAS-dependent transformation of cells did not influence cellular migration when fetal bovine serum (FBS) was used as a chemoattractant (Figure 3A–3C), invasion through a Matrigel barrier was dramatically stimulated by expression of HRAS G12V (Figure 3D–3F). In order to investigate the role of the carboxyl-terminal containing plasminogen receptors in invasion, we treated control and HRAS-transformed HEK-293 cells with ε-ACA or carboxypeptidase B. These treatments resulted in a significant loss in both RAS-dependent and RAS-independent invasion and this effect was mirrored when the plasmin activity of the invading cells was inhibited with aprotinin (Figure 3D). Oncogenic HRAS mediates the upregulation of MMP2 and MMP9 which play a key role in degrading the extracellular matrix [37]. Consistent with those reports, we observed that the broad-spectrum MMP inhibitor, GM6001 also inhibited both RAS-independent and RAS-dependent invasion (Figure 3D). This result is also consistent with the fact that plasmin activates several MMPs and this mechanism is important in plasmin induced invasion. These data highlight, for the first time, the significant role that plasminogen receptors play in RAS-dependent cellular invasion.

### Regulation of plasminogen receptors by oncogenic RAS

**Figure 4:**
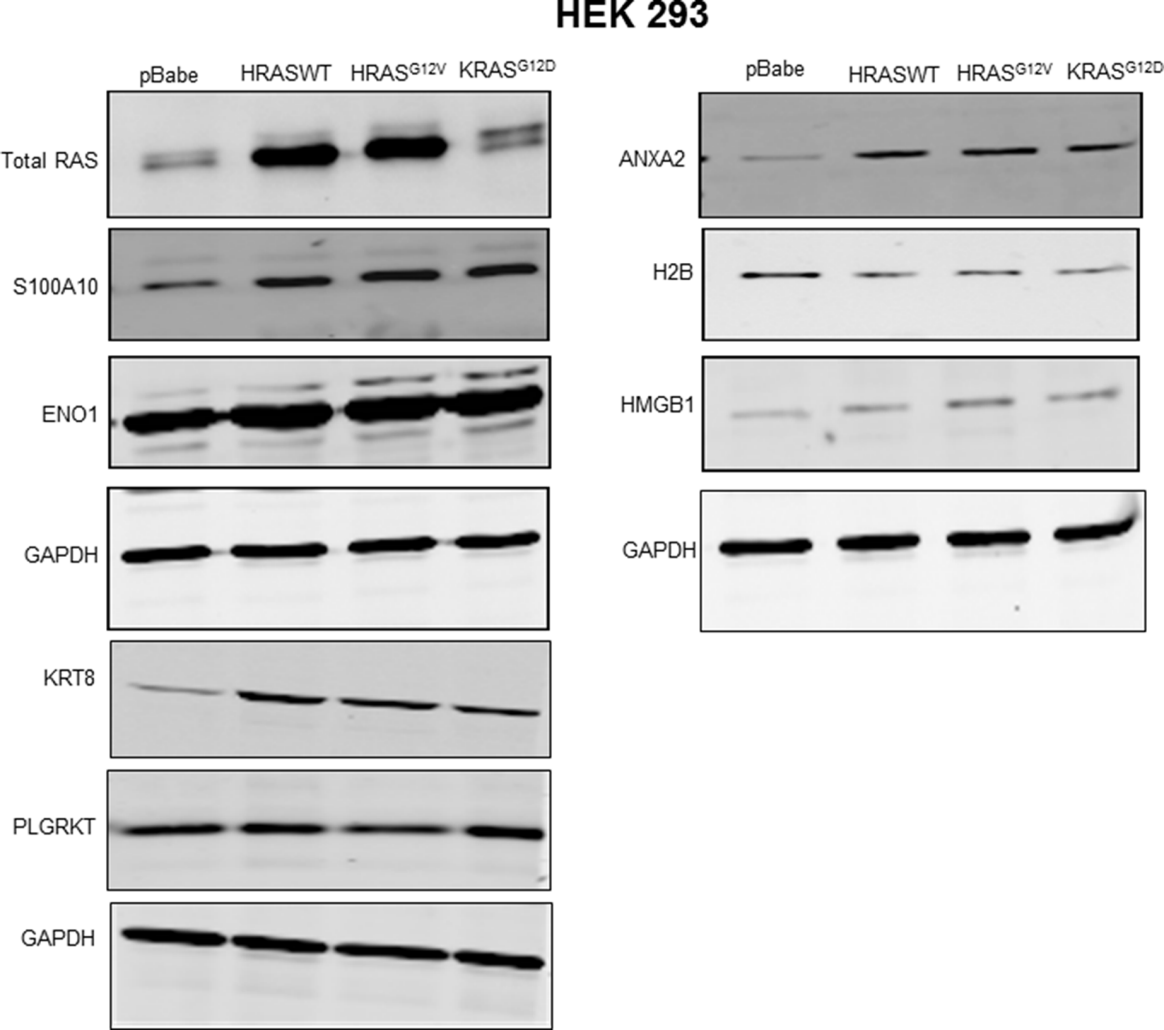
Regulation of plasminogen receptor expression by RAS Transfected HEK 293 cells expressing empty vector (pBabe control), wild type HRAS (HRAS WT), oncogenic HRAS G12V (HRAS^G12V^) or oncogenic KRAS G12D (KRASG12D) were selected and cultured for two weeks. The stably transfected cells were plated and cultured for two to three days, then lysed in lysis buffer containing protease and phosphatase inhibitors. Following lysis, the protein concentration was determined using Bradford reagent. The total protein (20–30 μg) was separated in 10–20% gradient gel, and transfered to a nitrocellulose membrane. The membrane was probed with primary antibodies as indicated and subsequently probed with IR-dye conjugated secondary antibodies, and scanned using the Li-COR Odyssey imager.

Plasminogen receptors are a heterogeneous group of cell surface proteins whose expression accounts for the total plasminogen-binding capacity of the cell with approximately 10^5^–10^7^ binding sites per cell [27, 46]. Of the plasminogen receptors, enolase-1 [43, 47], cytokeratin 8 [48, 49], HMGB1[45, 50], Plg-Rkt [51], S100A4 [52], S100A10 [29, 30] and histone H2B [53] are the most intensively studied. As shown in Figure 4, of these plasminogen receptors, only cytokeratin 8 and S100A10 (and its binding partner, ANXA2) were increased after oncogenic HRAS expression in HEK 293 cells. As observed for plasmin activity, it was noted that expression of wild-type HRAS or oncogenic KRAS also resulted in elevated levels of S100A10 and ANXA2 (Figure 4). However, the effect of RAS on cytokeratin 8 was not observed in all cell lines examined as 293T cells transfected with oncogenic *HRAS* failed to show an increased expression of cytokeratin 8, whereas S100A10 and annexin A2 expression were elevated in these cells (Supplementary Figure S3). Cytokeratin 8 expression has been reported on the surface of cancer cells and only in certain epithelial cells [48, 54–56] and to be upregulated in HRAS expressing epidermal cells [57]. Nonetheless, the loss of cytokeratin 8 expression rather than its upregulation is associated with metastasis and chemoresistance [58]. In contrast, S100A10, a carboxyl-terminal lysine-containing plasminogen receptor has been shown to account for the generation of as much as 90% of the cell surface plasmin generation [24, 33] and has been shown to play an important role in both tumor growth and metastasis [31, 32]. Therefore, considering the well-established role of S100A10 in plasmin generation we focused our subsequent studies on S100A10.

### Regulation of S100A10 expression by oncogenic RAS

**Figure 5:**
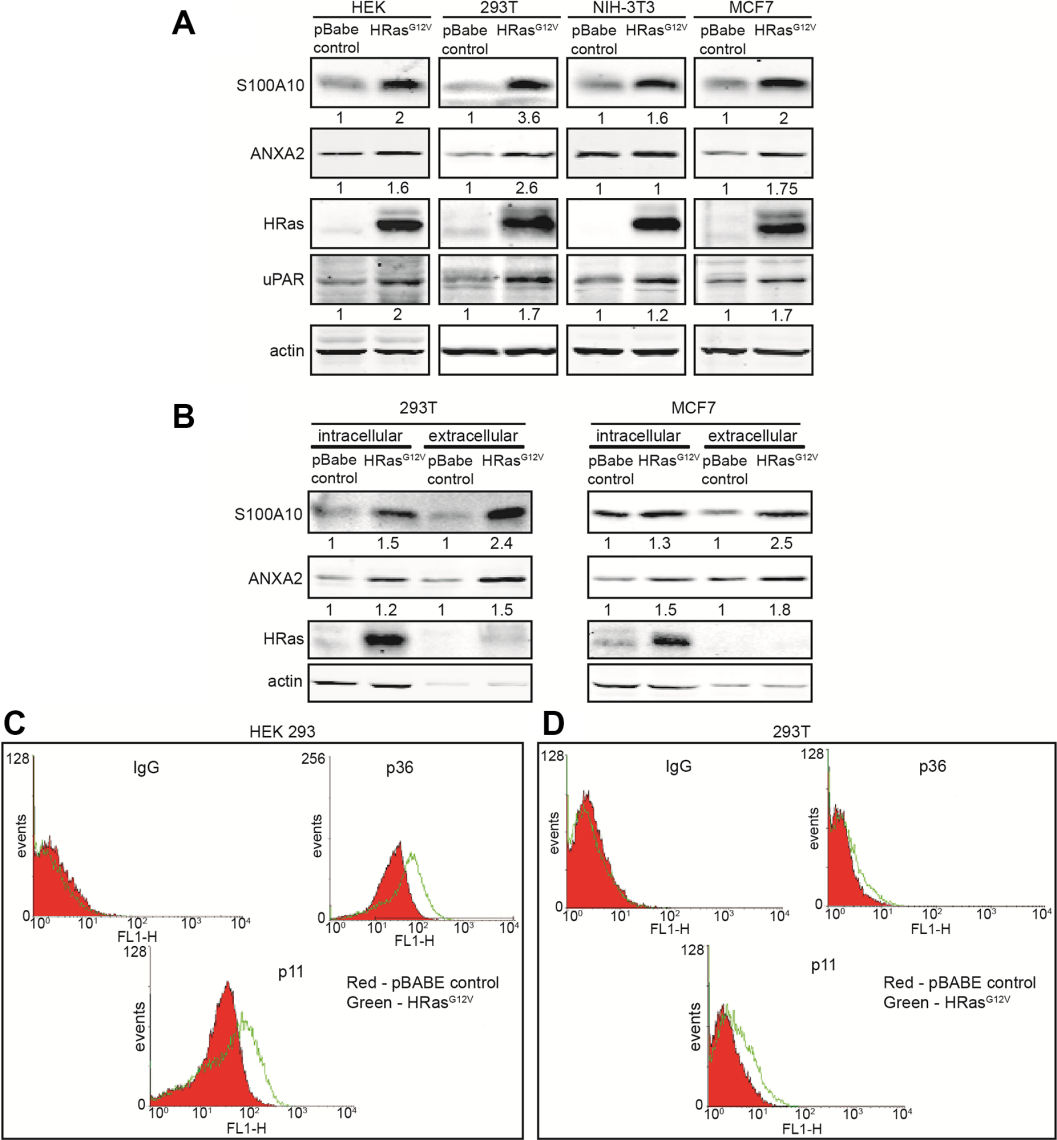
Oncogenic RAS stimulates S100A10 protein expression HEK 293, 293T, NIH-3T3 and MCF7 cells were transduced with either empty vector retrovirus (pBabe control) or oncogenic HRAS G12V expressing retrovirus (HRAS^G12V^). (**A**) 20 μg of each total cell extract was subjected to SDS-PAGE followed by western blotting with the antibodies indicated. (**B**) 293T and MCF7 pBabe control or HRASG12V cells were incubated with PBS containing 20 mM EGTA to elute the cell surface bound S100A10-ANXA2 complex. Cells were centrifuged at 1000 g for 5 minutes and elutes containing cell surface S100A10 were stored. Cell pellets containing intracellular S100A10 were lysed. 20 μg of each total cell extract and equivalent amounts of each eluted sample were subjected to SDS-PAGE followed by western blotting with the antibodies indicated. Quantification of protein bands was performed with the LI-COR Odyssey scanner software; (**C**) HEK 293 or (**D**)293T pBabe control or HRASG12V transduced cells were incubated with S100A10 and ANXA2 antibodies, after which a secondary FITC-labeled antibody was added. Cells were washed two times with PBS containing Ca2+ and S100A10 (p11) and ANXA2 (p36) cell surface protein expression was analyzed by FACS.

Our data suggested that S100A10 was regulated by oncogenic RAS and might be at least partially responsible for the oncogenic RAS-dependent increases in cellular plasmin generation. S100A10 is present on the cell surface as a heterotetrameric complex with its binding partner, ANXA2 which aids in tethering S100A10 to the membrane (reviewed in [59]). We observed that both S100A10 and its binding partner, ANXA2, were upregulated in a variety of cells expressing oncogenic HRAS (Figure 5A). This increase in total levels of S100A10 and ANXA2 was observed on the extracellular surface of HRAS^G12V^ expressing cells (Figure 5B–5D).

One of the most well established mouse models of pancreatic cancer is the inducible-Ras (iKRas) mouse model. This model system allows tissue-specific, inducible and reversible expression of mutant KRas [60]. The iKRas mice develop pancreatic cancer that is dependent on sustained iKRas activity. Using the pancreatic cancer cells isolated from iKRas tumors we interrogated the relationship between iKRas and S100A10. We observed that activation of the expression of iKRas in these cells resulted in a dramatic increase in expression of S100A10 (Supplementary Figure S4).

Expression of oncogenic RAS is a common feature of many cancers particularly pancreatic, lung, colon, sarcomas among others. In order to determine if there was a relationship between oncogenic RAS expression and S100A10 levels in clinically relevant cancer cells, we analyzed by western blotting, a panel of different cancer cell lines of which some express oncogenic RAS (Supplementary Figure S5). Our results showed a significant correlation between oncogenic RAS expression and S100A10 levels. For example, we observed that the oncogenic KRAS expressing breast cancer cell line MDA MB 231 showed much higher levels of S100A10 compared to MCF7 breast cancer cells (that do not express oncogenic RAS).

To further investigate the S100A10 levels in clinically relevant cancer cell lines, we depleted KRAS from A549 (lung cancer) and MiaPaca2 (pancreatic cancer) cells and analysed S100A10 expression by western blotting (Supplementary Figure S6A, S6B). These results showed a significant downregulation of S100A10 in KRAS-depleted cells compared to control cells. Interestingly, within the pools of cells that did not show downregulation of KRAS, we did not observe decreased expression of S100A10. These results further confirm the regulation of S100A10 protein levels by oncogenic RAS.

**Figure 6:**
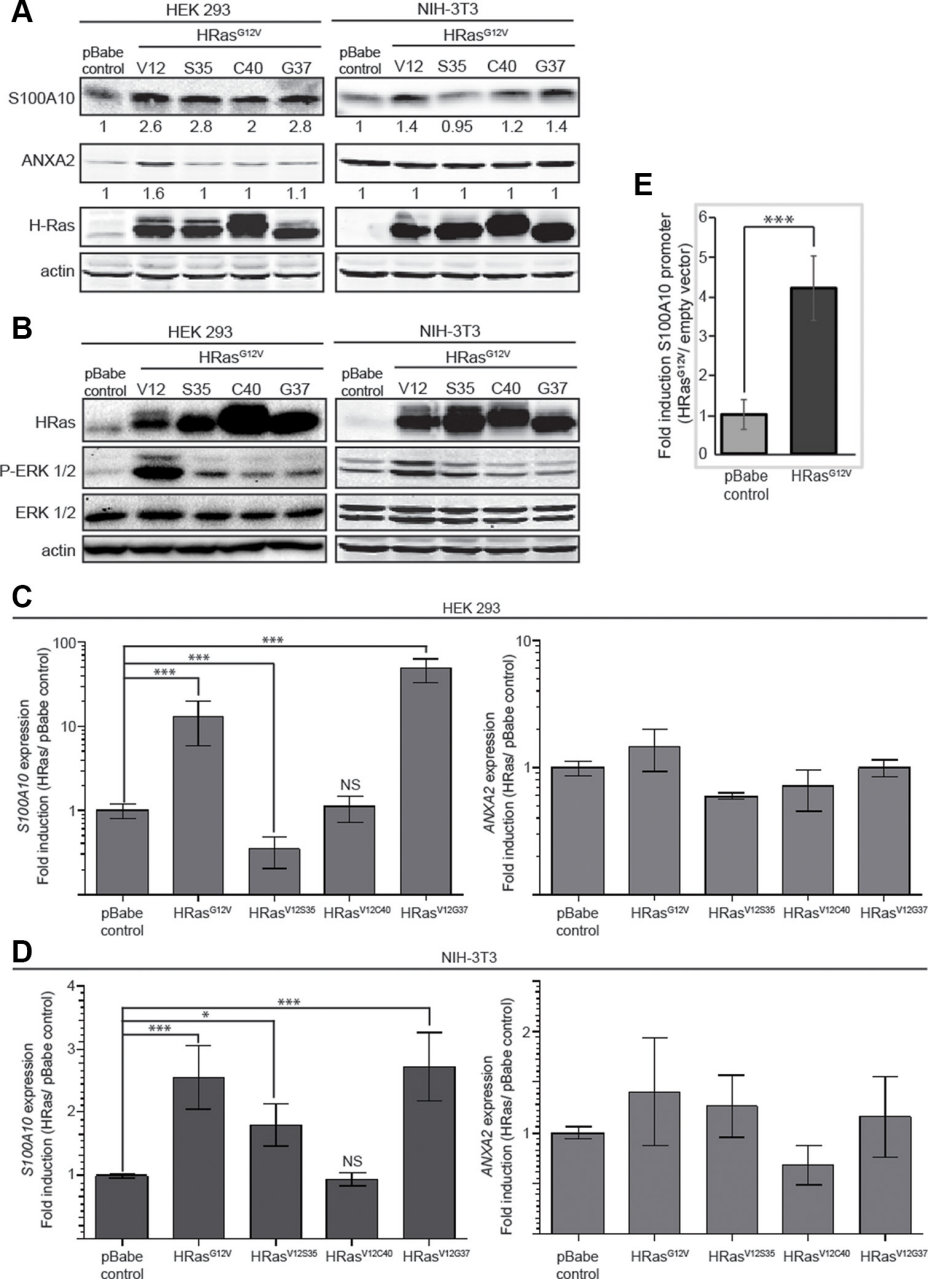
Mechanism of regulation of S100A10 expression HEK 293 and NIH-3T3 cells were transduced with either empty vector retrovirus (pBabe control), oncogenic HRAS G12V expressing retrovirus (HRASG12V), oncogenic HRAS S35 mutant (HRASV12S35), oncogenic HRAS C40 mutant (HRASV12C40) or oncogenic HRAS G37 mutant (HRASV12G37). (**A**) 20 μg of each total cell extract was subjected to SDS-PAGE followed by western blotting with the antibodies indicated. (**B**) 20 μg of each total cell extract from HEK 293 or NIH-3T3 pBabe control, HRAS^G12V^, HRAS ^V12S35^, HRAS ^V12C40^ or HRAS ^V12G37^ was subjected to SDS-PAGE followed by western blotting with the antibodies indicated; (**C**, **D**) RNA from HEK 293 (C) or NIH 3T3 (D) cells transduced with empty vector retrovirus (pBabe control), oncogenic HRAS G12V expressing retrovirus (HRASG12V), oncogenic HRAS S35 mutant (HRASV12S35), oncogenic HRAS C40 mutant (HRASV12C40) or oncogenic HRAS G37 mutant (HRASV12G37) was isolated by Trizol RNA extraction; cDNA was generated and the mRNA expression level for ANXA2 and S100A10 was measured by qRT-PCR as described in materials and methods. The mRNA expression levels were normalized to GAPDH mRNA using the 2^−ΔΔCT^ method and are represented as ratio of RAS transformed over non-transformed cells. Error bars represent the standard deviation obtained from at least three independent studies performed with triplicate samples. (**E**) HEK 293 pBabe control and HEK 293 HRAS G12V expressing cells were co-transfected with pGL4.22-S100A10 full-length promoter and pRSV-Renilla (transfection control) at a 50:1 ratio. Luciferase promoter assay was performed according to the manufacturer's instructions (Dual Luciferase promoter assay, Promega). Error bars represent the standard deviation obtained from five independent experiments with at least triplicate samples each. Statistical analysis was performed by Student's *t*-test.

A region of the RAS protein referred to as the effector domain has been shown to be essential for the interaction between RAS-GTP and several of its downstream effectors. Effector loop mutations alter the ability of HRAS to activate the Raf1 (V12S35RAS) vs PI3K (V12C40RAS) vs RalGDS (V12G37RAS) signaling pathways and preferentially activate one pathway but not the others [61]. The effector domain mutants of oncogenic HRAS were tested for a possible role in the regulation of S100A10 protein levels. As shown in Figure 6A, S100A10 protein expression was activated by all three effector mutants whereas annexin A2 protein levels were unaffected by the effector mutants in HEK293 cells. In contrast, in NIH3T3 cells, the V12S35RAS mutant that selectively activates Raf1 failed to increase S100A10 protein levels (Figure 6B). This suggested that the PI3K and RalGDS pathways contributed to enhanced S100A10 protein levels in both cell lines but only in the HEK 293 cells did the Raf1 pathway contribute to expression of S100A10 protein levels. This observation was consistent with other studies suggesting that RAS signaling exhibits significant cell context variations [62].

Semi-quantitative RT-PCR (qRT-PCR) analysis showed that oncogenic RAS activated *S100A10* gene expression but not *annexin A2* gene expression (Figure 6C, 6D). Furthermore, in both HEK 293 and NIH 3T3 cells, HRASV12G37 a mutant that predominately activates the RAS/RalGDS pathway, stimulated *S100A10* gene expression, implicating the importance of the RalGDS pathway in the regulation of *S100A10* expression (Figure 6B, 6C). To further verify that oncogenic RAS affected transcription of the *S100A10* gene, HEK 293 cells were transfected with the luciferase reporter construct pGL4-S100A10. HEK 293 V12HRAS cells showed a four-fold increase in p11 promoter activity compared to HEK 293 pBABE cells indicating that RAS-responsive elements are contained within the *S100A10* promoter (Figure 6E).

### S100A10 plays a key role in oncogenic RAS-dependent plasmin generation

**Figure 7:**
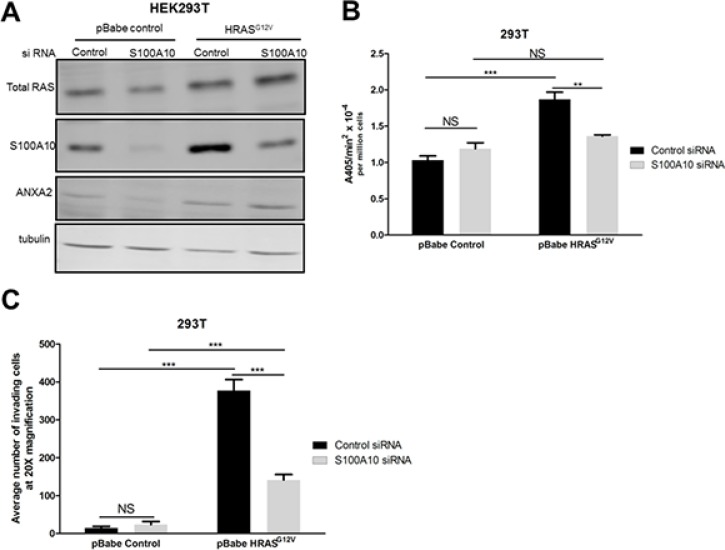
The S100A10 plasminogen receptor plays a major role in oncogenic RAS-dependent plasminogen activation (**A**) 293T cells stably expressing empty vector (pBabe control) or oncogenic HRAS G12V (HRASG12V) were transfected with 4 μM of pre-designed siRNA (Ambion) specific for S100A10 (S100A10 siRNA) and a non-silencing siRNA control (siRNA control) using Lipofectamine 2000 transfection reagent as per manufacturer's instructions (Invitrogen). Cell lysates were prepared 48 hours after transfection, and total levels of S100A10 and RAS were examined by Western blotting. (**B**) 48 hours after transfection, 25,000 cells were plated in 96 well plates and incubated overnight. Cells were washed 3 times with incubation buffer (Hanks balanced salt solution containing 3 mM CaCl_2_ and 1 mM MgCl_2_) and incubated with 0.5 μM glu-plasminogen for 20–30 minutes before the addition of 500 μM plasmin substrate S2251. The rate of plasmin generation was measured from the A405 nm vs min^2^ progress curves (*N* = 4). (**C**) 48 hours after transfection, 100,000 cells were plated on the upper chamber of matrigel coated inserts (BD Biosciences) and incubated in serum free medium containing 0.5 μM plasminogen for 48 hours. The lower chamber contained medium with 10% fetal bovine serum (FBS) as a chemoattractant. Invading cells were fixed with methanol and stained with Hematoxylin and Eosin. Cells were quantified by manual counting using a light microscope (20X). Data are expressed as mean number of cells invading per 20× field (5 fields/experimental condition) in duplicates. The plot is representative of three independent experiments. Statistical analysis was performed by two-way ANOVA with Tukey test of significance.

Although our data suggested that RAS-dependent transformation of cells increased plasmin generation and cellular invasiveness concomitant with an increase in S100A10 levels it was unclear if this relationship was causal or coincidental. This question was approached by depleting S100A10 levels from *RAS*-transformed cells and measuring both plasmin generation and cellular invasiveness. Depletion of S100A10 did not affect the levels of other plasminogen receptors (Supplementary Figure S7). We observed that the depletion of S100A10 (Figure 7A) resulted in a significant loss of both *RAS*-dependent increases in plasmin generation (Figure 7B) and cellular invasiveness (Figure 7C), therefore establishing that S100A10 is responsible for much of the increases in plasmin generation and cellular invasion that are activated upon RAS transformation.

## DISCUSSION

**Figure 8:**
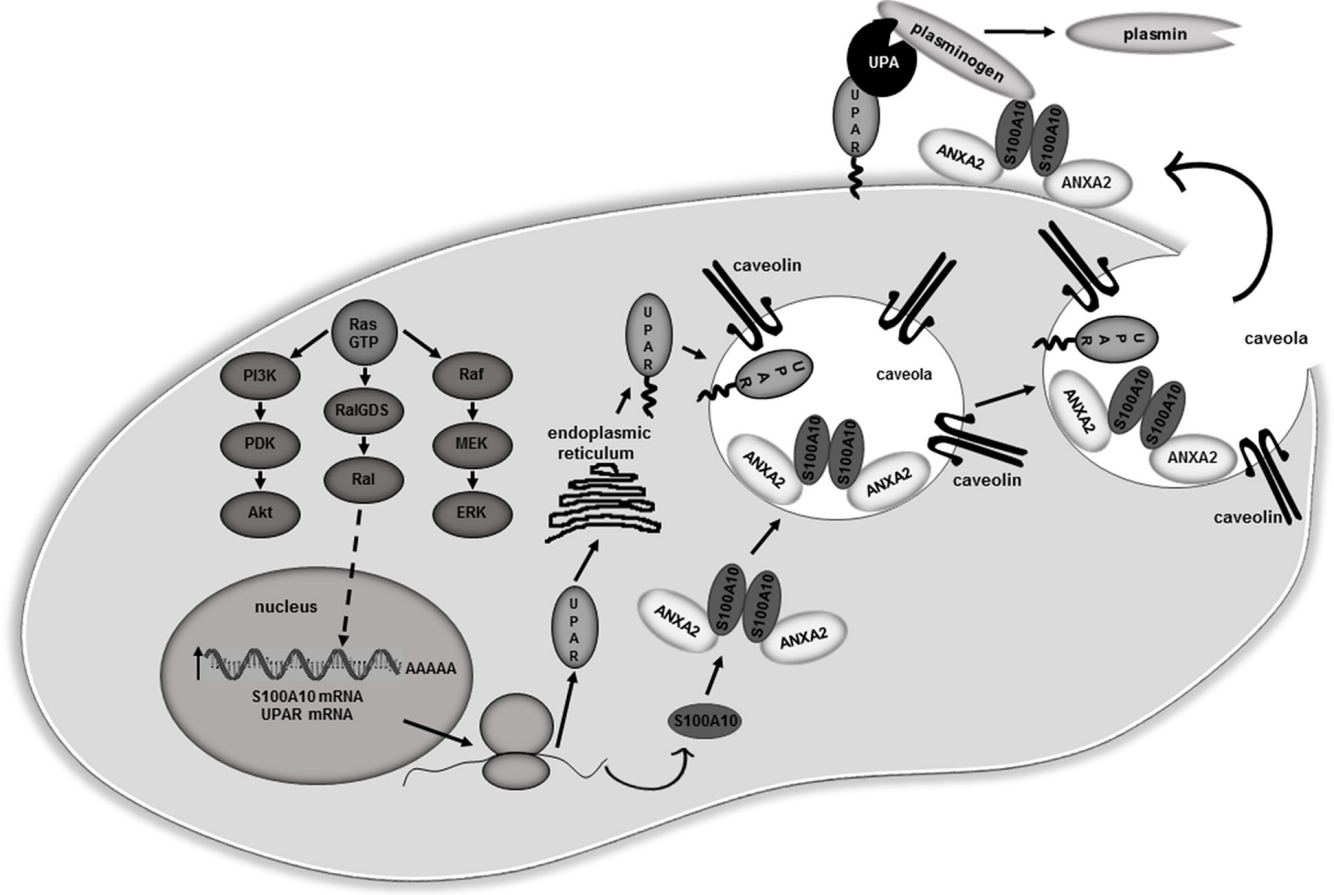
Proposed model for S100A10 dependent regulation of plasmin activity in RAS transformed cells Oncogenic RAS up-regulates S100A10 and uPAR mRNA levels via the Ral-GDS/Ral pathway leading to increased expression of these proteins. Newly synthesized uPAR protein undergoes glycosylation in the endoplasmic reticulum and is shuttled to the cell surface. Newly synthesized S100A10 associates with its binding partner ANXA2 forming the ANXA2-S100A10 heterotetramer (AIIt). Both uPAR and AIIt localize in caveolae lipid rafts at the cell surface. Secreted uPA forms a complex with uPAR while the plasminogen present in the extracellular fluid forms a complex with the S100A10 subunit of AIIt. Plasmin generation is stimulated due to co-localization of the uPA/uPAR and plasminogen/AIIt complexes. The increased plasmin generation contributes to the overall proteolytic activity of the RAS-transformed cells which results in increased cellular invasiveness

The first committed step in metastasis, the departure of tumor cells from a solid malignant tumor is controlled by three events, namely: 1) the attachment to and interaction of the tumor cells with components of the ECM and basement membrane: 2) the activation of tumor cell migration and: 3) the activation of local proteolysis. Increased extracellular protease activity is one of the distinguishing features of metastatic cells. Proteases facilitate the invasion of malignant/metastatic cells by promoting the degradation of basement membranes and stromal ECM thereby facilitating their intravasation into the blood and/or lymph vessels. Evidence has accumulated that different types of tumor-associated proteases as well as their inhibitors and receptors are all involved in tumor invasion and metastasis. However, of the many oncogenic proteins that drive transformation, mutations that lead to constitutively active RAS and its associated activation of downstream signaling molecules have been implicated in approximately 30% of all human cancers [63]. In this regard, oncogenic RAS-mediated upregulation of MMP2, MMP9, and cathepsin B has been shown to be particularly important in stimulating cancer cell proteolytic activity and invasion [37]. The introduction of oncogenic RAS into cells increases extracellular protease activity in general and the expression of uPAR in particular [64–69]. In the current study, we investigated the relationship between expression of oncogenic RAS and activation of plasmin proteolytic activity at the cell surface of cancer cells. We observed that expression of oncogenic RAS results in a substantial increase in cellular plasmin generation in a number of cell lines suggesting that this phenomenon is a common feature of RAS-transformed cancer cells. We also demonstrate the fundamental role of the plasminogen receptor, S100A10 in RAS-dependent cellular plasmin generation. A model conceptualizing our data is provided in Figure 8.

The uPA and uPAR are expressed in almost every malignant solid tumor studied to date, while most normal tissues express little or none. The uPA is overexpressed by most aggressive tumor phenotypes and the expression of uPA and uPAR is regulated by both growth factors and oncogenes. Clinical studies have revealed that high expression levels of uPA and uPAR correlate with poor patient prognosis and outcome. It is well established that uPA stimulates cellular proteolytic activity by converting the zymogen plasminogen into the active serine protease plasmin. It was initially reported that both uPAR levels and laminin degradation were decreased in HCT 116 cells in which the KRAS oncogene had been ‘knocked out’ by homologous recombination [35]. More recently, Quigley and coworkers highlighted the critical importance of the uPA/plasmin system in the ability of tumor cells to intravasate into the blood vessels. They directly demonstrated that inhibition of plasmin activity resulted in a loss in tumor cell invasiveness *in vivo* [11]. These and other studies have presented the possibility that the observed increased plasmin activity demonstrated by cells expressing oncogenic RAS results from RAS-dependent increases in uPA and/or uPAR. However, we observed that compared to control cells, RAS-transformed cells had a greater capacity to generate plasmin in the presence of saturating amounts of exogenous uPA. Since the control and RAS-transformed cells had equivalent amounts of uPA, and under these conditions the RAS-transformed cells still generated much more plasmin than the control cells, the simplest explanation for this result is that RAS-transformed cells upregulate their plasminogen receptors which then results in a substantial increase in plasmin generation. This suggestion contrasts with other models in which increased secretion of uPA accounts for increased plasmin generation by RAS-transformed cells. Clearly, identification of the plasminogen receptors that participate in RAS-dependent increases in plasmin generation is of importance in understanding the molecular mechanisms involved in metastasis.

Our observation that ε-ACA and carboxypeptidase B, two treatments that are known to block plasminogen binding to the cell surface [42, 43] resulted in the total loss of RAS-dependent plasmin generation by several cancer cell lines is a paradigm shift in that it establishes that plasminogen receptors are necessary for plasmin generation by RAS-transformed cancer cells. Furthermore, our data also suggests that plasminogen receptors that possess a carboxyl-terminal lysine play the key role in RAS-dependent plasmin generation. Numerous cell surface molecules are capable of binding plasminogen, thereby facilitating its conversion into plasmin [43]. The majority of these cell membrane plasminogen receptors possess carboxyl-terminal lysines, which allow them to function as plasminogen receptors via direct binding of their carboxyl-terminal lysine to the lysine-binding sites located in the kringle domains of plasminogen. The identity of the key plasminogen receptors participating in the activation of uPA-dependent plasmin generation have not been determined. In order to identify the plasminogen receptors that participate in RAS-dependent plasmin generation, we investigated if transformation of cells with oncogenic RAS affected the expression of the well characterized plasminogen receptors, namely, enolase, histone H2B, cytokeratin 8, PlgR_kt_, HMGB1, S100A10 and S100A4. Our study established, for the first time, that only cytokeratin 8 and S100A10 protein levels are increased in HEK 293 cells expressing oncogenic RAS. In contrast, S100A10 but not cytokeratin 8 expression was increased in 293T cells. However, unlike S100A10, the loss of cytokeratin 8 is known to facilitate the increased migration and invasiveness of epithelial cancer cells [58]. Therefore, it is difficult to envision a role for cytokeratin 8 in invasion and metastasis.

Depletion of S100A10 in oncogenic RAS expressing cells established that S100A10 is a key plasminogen receptor involved in plasmin activation and in the promotion of the enhanced invasive phenotype observed in RAS transformed cells. Although other reports had previously identified the uPA/uPAR system as important effectors of RAS induced cell invasiveness, the plasminogen receptor(s) involved in cell surface plasmin generation were still unknown. We had previously shown that the S100A10/ANXA2 heterotetramer co-localizes with uPAR at the cell surface. Here we show for the first time that S100A10 plays a main role in RAS-induced plasmin formation and invasion.

We also sought to determine the mechanism by which oncogenic RAS up-regulates S100A10 protein. Since we observed that *S100A10* mRNA was upregulated and the *S100A10* promoter was stimulated by oncogenic RAS transformation, we have concluded that S100A10 is transcriptionally regulated by oncogenic RAS. When in the GTP-bound state, RAS is capable of binding to several different established and potential effector proteins, including members of the Raf, PI(3)K and RalGDS families. To examine which of these pathways regulated *S100A10* expression, we transfected cells with *RAS* effector loop mutants. Certain point mutations in the RAS effector loop region (amino acids 32 to 40 in HRAS) render HRAS defective for binding specific effector proteins while remaining competent for binding and activating others, albeit at lower than wild-type efficiency [61, 70]. We observed that the HRASV12G37, a mutant that predominately activates the RAS/RalGDS pathway, stimulated *S100A10* gene expression, implicating the importance of the RalGDS pathway in the transcriptional regulation of S100A10. Interestingly, the RalGEF pathway has been shown to significantly contribute to RAS-initiated metastasis in cellular systems [71]. S100A10 protein level was also increased in HRASV12C40 expressing cells even though this mutant HRAS did not induce *S100A10* gene transcription. This result suggests that the PI3K pathway might be involved in increasing the stability of the S100A10 protein by a still unknown mechanism. It is therefore reasonable to speculate that activation of S100A10-dependent plasmin generation may contribute to the metastatic phenotype observed in these model systems.

S100A10 was originally identified as a potent activator of plasmin generation *in vitro* [29, 30] that bound tPA (0.45 μM), plasminogen (1.8 μM) and plasmin (0.36 μM) [72]. Subsequent studies showed that S100A10 could account for between 50–90% of the plasmin generation by a number of cells (reviewed in [33]). S100A10 is overexpressed in animal tumor model systems and in human cancer, where it correlates with a poor prognosis and chemotherapeutic resistance [73–80]. In various animal tumor model systems, the inhibition of S100A10 expression, or activity, decreases tumor growth and metastasis [31, 32]. Mutations of the *KRAS* gene occur in over 90% of pancreatic carcinomas. S100A10 levels are increased in patients with pancreatic cancer [80]. Furthermore, using both immunohistochemical analysis and quantitative proteomics of patient tumors it has been demonstrated that S100A10 levels were unchanged in pancreatitis, but dramatically increased in late stage pancreatic intraepithelial neoplasia lesions and in pancreatic ductal adenocarcinoma lesions. These patient data highlight the potential importance of S100A10 in KRAS-driven pancreatic cancer. Interestingly, we also observed that activation of KRas in mouse pancreatic cancer cells resulted in the increased expression of S100A10 (Supplementary Figure S4). Aberrant activation of the RAS pathway due to mutations in the RAS GTPase activating protein is ubiquitous in human liver cancer and this unrestrained activation of wild-type RAS is a key feature of hepatocarcinogenesis [81]. In the present report we show a significant relationship between oncogenic KRAS expression and enhanced levels of S100A10 in a panel of different human cancer cell lines. It was striking to observe that cells derived from cancers that show high mutational rates for *KRas*, such as colon (HCT 116), lung (A549) and pancreatic (MiaPaca2) cancers, have high levels of expression of S100A10 (Supplementary Figure S5). Interestingly, we also observed that the breast cancer cell line MDA MB 231 that expresses oncogenic KRAS (which is not a commonly acquired mutation in this type of cancer) shows higher levels of expression of S100A10 compared to the breast cancer cell line MCF7 that express WT KRAS (Supplementary Figure S5). To further confirm that KRAS activity is important for the regulation of S100A10 expression in cancer cells derived from human tumors, we depleted KRAS from A549 and MiaPaca2 cells. This data showed a significant downregulation of S100A10 in these cells (Supplementary Figure S6), supporting the concept that KRAS activity plays a key role in regulating S100A10 levels in cancer cells.

S100A10 is also responsible for the hyperfibrinolytic syndrome exhibited by patients with acute promyelocytic leukemia (APL). In this regard, S100A10 was shown to be regulated by the PML-RAR oncoprotein, the causative agent responsible for APL [34]. Collectively, these results suggest that the regulation of S100A10 by the oncogenes, PML-RAR and RAS plays an important role in oncogenesis.

Recent developments in cell capture technology have allowed, for the first time, the isolation of circulating tumor cells from cancer patients [82]. This approach facilitated identification of genes that are directly activated during the initial stages of metastasis, namely intravasation. Interestingly, this group reported the activation of the *S100A10* gene during intravasation of breast cancer cells and showed that *S100A10* was one of only 170 genes activated during this process. Furthermore, of all the well characterized plasminogen receptor genes, only the *S100A10* gene was identified in this analysis, *in vivo*. This study therefore highlighted *S100A10* as a key gene that is activated during the early stages of metastasis. Our current study sheds new light on the role of S100A10 in metastasis by demonstrating that oncogenic RAS activates the *S100A10* gene and that S100A10 is critical for this RAS-dependent increases in cellular plasmin generation and invasiveness.

## MATERIALS AND METHODS

### Cell culture, transfections and cell lines

MCF-7, MDA MB 231, HEK 293, Phoenix, A549, MiaPaca2, HT1080, HCT 116, NIH 3T3 and 293T cell lines were obtained from ATCC and maintained in Dulbecco's modified Eagle's medium (Hyclone) supplemented with 10% fetal bovine serum (FBS) and 100 U/ml of penicillin/streptomycin, in a humidified incubator in an atmosphere of 5% CO_2_ at 37°C. Cells were regularly tested for mycoplasma. For retrovirus production, Phoenix cells were transfected with 4 μg of the pBabe-puro HRAS V12 and HRAS loop mutants plasmids described below using 12 μl of L-lipofectamine 2000 transfection reagent (Thermofisher) according to the manufacturers' instructions. 48 hours after transfection the target cells were infected with Phoenix supernatants and selected with 2 μg/ml of puromycin. Cells were regularly tested for mycoplasma. A549 or MiaPaca2 cells plated in 60 mm plates were transfected with 3 μg pKRAS-gRNA-px459-V2 using 6 μl of jetPRIME reagent (Polyplus) according to the manufacturers' instructions. 48 hours after transfection the cells were selected with 5 μg/ml of puromycin. Serial dilutions were performed to obtain A549 or MiaPaca2 KRAS subpopulations.

### Plasmids

The plasmids pBabe-puro (Addgene # 1764) were a gift from Hartmut Land and Jay Morgenstern, pBabe-puro HRAS V12 (Addgene # 39526), were a gift from Julian Downward and HRAS V12 (Addgene # 9051) was a gift from William Hahn, pbabe - KRAS G12D (Addgene # 58902) was a gift from Channing Der pBabe-puro HRAS V12 S35 (Addgene # 18746) was a gift from Jay Morgenstern, pBabe-puro HRAS V12 C40 (Addgene # 18747) and pBabe-puro HRAS V12 G37 (Addgene # 18745) was a gift from Scott Lowe S100A10 siRNA was purchased as a pre-designed sequence from Ambion (4392420). The pKRAS-gRNA1-px459-V2 plasmid was made by cloning the annealed oligos: 5′- CAC CGA ATA TAA ACT TGT GGT AGT-3′ and 5′-AAA CAC TAC CAC AAG TTT ATA TTC-3′ into px459-V2 from Addgene (62988) and the pKRAS-gRNA2-px459-V2 was made by cloning the annealed oligos: 5′-CAC CGA AAC TTG TGG TAG TTG GAG C-3′ and 5′-AAA CGC TCC AAC TAC CAC AAG TTT C-3′ into px459-V2 from Addgene. The pGL4.22- S100A10-promoter luciferase reporter plasmid was constructed by cutting the S100A10-promoter from the pCAT-S100A10-promoter plasmid and cloning it into the Kpn I/Sac I restriction sites of pGL4.22 vector (Promega).

### Antibodies

The following antibodies were used for western blot analysis: ANXA2 antibodies: 610069 (BD Transduction laboratories), D1/274.5 hybridoma (made in house); S100A10 antibodies: 610071 (BD Transduction laboratories), sc-81153 (SCBT); actin antibodies: (AC-40), A3853 (SIGMA), sc-1615 (SCBT); β-tubulin antibody (H-235), sc-9104 (SCBT); HRAS antibody, sc-520 (SCBT), RAS antibody, clone RAS10, 05-516 (Millipore); KRAS antibody, H00003845-M01 (AbNova); H2B antibody, ab52599 (Abcam); HMGB1 antibody, H9537 (Sigma); Cytokeratin 8 antibody, ab53280 (Abcam); non-neuronal enolase antibody, ab54979 (Abcam); GAPDH antibody, sc-25778 (SCBT); uPAR antibody, 399R (American Diagnostica); ERK 1/2 antibodies: sc-135900 (SCBT), 9102 (Cell Signaling); P-ERK 1/2 antibodies: 9101S (Cell Signaling), sc-377400 (SCBT), ERK 1/2 antibody: sc-514302 (SCBT). The Plg R_KT_ antibody was a kind gift from Dr. Lindsey Miles (Scripps Research Institute, San Diego, California).

### Western blot analysis

For western blot analysis, 20–25 μg of each cell lysate, unless noted otherwise, was subjected to SDS-PAGE, transferred onto a nitrocellulose membrane, incubated with appropriate antibodies and visualized using a LI-COR Odyssey scanner (LI-COR Biosciences) or a ChemiDoc XRS+ system (Bio-Rad). Quantification of protein bands was done using the LI-COR Odyssey scanner software or Image J software as indicated in the figure legends.

### Elution of the S100A10-ANXA2 complex from the cell surface

Cells were incubated with PBS containing 20 mM EGTA in order to elute the cell surface bound S100A10-ANXA2 heterotetramer (AIIt). The AIIt binds to the cell surface via calcium-dependent phospholipid binding domains existent in the ANXA2 moiety of the complex. Cells were centrifuged at 1000 g for 5 minutes and elutes containing cell surface S100A10 and ANXA2 were stored at −80°C. Cell pellets containing intracellular S100A10 and ANXA2 were lysed. 20 μg of each total cell extract and equivalent amounts of each eluted sample were subjected to SDS-PAGE followed by western blotting with the antibodies indicated.

### FACS analysis

To assess the surface expression of S100A10 and ANXA2, cells were Fc blocked with rabbit IgG and then incubated with antibodies against S100A10 and ANXA2 in PBS containing 1% FBS for 1 hour at 37°C, 5% CO_2_. Cells were washed two times with PBS containing Ca^2+^ and incubated with goat anti-mouse secondary antibody tagged with fluorescein isothiocyanate (FITC) (Sigma Aldrich) for 1 hour at 37°C, 5% CO_2_. Cells were washed two times with PBS containing Ca^2+^ and S100A10 and ANXA2 cell surface protein expression was analyzed by FACS, using a FITC filter. FACS data was analysed using the Winmdi software.

### qRT-PCR

RNA from cells transduced with empty vector retrovirus (pBabe), HRAS V12 or HRAS loop mutants expressing retroviruses was isolated by Trizol RNA extraction according to the manufacturer's instructions. Briefly, each plate was gently washed with 300 μl of Trizol after which the Trizol/cell mixture was transferred onto an Eppendorf tube. 100 μl of chloroform was added to the tube, shaken vigorously for 15 seconds, and incubated for 10 minutes at room temperature. The resulting mixture was centrifuged at 12,000 × g for 15 minutes at 4°C. The aqueous phase was transferred onto a fresh tube and 250 μl of isopropanol was added. The tube was inverted a few times and incubated for 10 minutes at room temperature. After what samples were centrifuged at 12,000 × g for 10 minutes at 4°C. The RNA pellet was washed with 300 μl of 75 % ethanol and resuspended with 100 μl of MiliQ water. cDNA was generated using superscript II (Invitrogen) or NZY M-MuLV First-Strand cDNA Synthesis Kit (Nzytech) according to the manufacturers' instructions and the mRNA expression level for ANXA2, S100A10 or GAPDH was measured by qPCR using Quanti-fast Sybr Green Master mix (Qiagen) or NZYSpeedy qPCR Green Master Mix (2x) (Nzytech) according to the manufacturers' instructions. The reaction was carried out using the MX3000P qRT-PCR machine or CFX96 Touch™ Real-Time PCR Detection System (Bio-Rad). The mRNA expression levels were normalized to GAPDH mRNA using the 2^−ΔΔCT^ method (Livak KJ, Schmittgen TD) and are represented as ratio of RAS transformed over non-transformed cells. Error bars represent the standard deviation obtained from at least three independent experiments performed in triplicates. The following primers were used for qPCR: Human genes: ANXA2 Fwd: 5′-CTC TAC ACC CCC AAG TGC AT-3′; ANXA2 Rev: 5′-TCA GTG CTG ATG CAA GTT CC-3′; S100A10 Fwd: 5′-AAA TTC GCT GGG GAT AAA GG-3′; S100A10 Rev: 5′-AGC CCA CTT TGC CAT CTC TA-3′; GAPDH Fwd: 5′-GAG TCA ACG GAT TTG GTC GT-3′; GAPDH Rev: 5′-TTG ATT TTG GAG GGA TCT CG-3′. Mouse genes: S100A10 Fwd: 5′-GAC CTG AGA GTG CTC ATG GA-3′; S100A10 Rev: 5′- CCG CCA CTA GTG ATA GAA AGC-3′; ANXA2 Fwd: 5′- CAG CAG TCA AGA CCA AAG GA-3′; ANXA2 Rev: 5′- GGC CAG ATA AGG CTG ACT TC-3′; GAPDH Fwd: 5′- ACA ACT TTG GCA TTG TGG AA-3′; GAPDH Rev: 5′- GAT GCA GGG ATG ATG TTC TG-3′.

### Plasmin activation assay

Cells were plated in 96 wells plate and washed three times with incubation buffer (Hanks balanced salt solution containing 3 mM CaCl_2_ and 1 mM MgCl_2_) Cells were then incubated with 0.5 μM glu-plasminogen with or without the plasmin inhibitors, aprotinin (2.2 μM, Pentapharm) or ε-aminocaproic acid (ε-ACA) (100 mM, synthetic inhibitor of the plasminogen system, SIGMA) or CpB (5 U/ml, SIGMA) or the broad specificity MMP inhibitor GM6001 (25 μM, Millipore), for 10 minutes before the addition of 250 μM plasmin chromogenic substrate, S2251 (Chromogenix, Diapharma Group). Plasmin activity was measured spectrophotometrically (405 nm) taking readings every minute for 2 hours. *N* = 6. Time course data are analyzed according to the equation describing the rate of *p*-nitroanilide (*p*-NA) production *A*405 nm = *B* + *Kt*2, where *K* is the rate constant for the acceleration of *p*-NA generation and *B* is the *y*-intercept. Under our experimental conditions, *K* is proportional to the initial rate of plasmin formation from plasminogen.

### Invasion/migration assays

10^5^ cells were plated on the upper chamber of Matrigel (MTG)-coated chambers (BD Biosciences) (invasion) or non-coated chambers (migration) and incubated in serum free medium containing 0.5 μM glu-plasminogen, with or without the plasmin inhibitor aprotinin, or ε-amino caproic acid (ε-ACA) or the broad specificity MMP inhibitor GM6001. Alternatively, some cells were also incubated with carboxypeptidase B (CpB) before plating as described previously [83]. The lower chamber contained medium with 10% fetal bovine serum (FBS), as a chemoattractant. Cells were incubated at 37°C and 5%CO_2_ for 24 hours. Invading cells were quantified according to the manufacturer's instructions. Data are expressed as mean number of cells per 40X field (10 fields/experimental condition) plus or minus StdDev of 4 independent wells. Statistical analysis was performed using the Student *t* test or using two-way ANOVA as indicated.

### Promoter assays

Cells were co-transfected with S100A10 full-length promoter in pGL4.22 backbone and pRSV-Renilla (transfection control) at a ratio of 50:1. Luciferase promoter assay was performed according to the manufacturer's instructions (Dual Luciferase promoter assay, Promega). Error bars represent the standard deviation obtained from five independent experiments with at least triplicate samples each.

### Depletion of S100A10 in HRAS V12 expressing cells

Pre-designed S100A10 siRNA sequences and a siRNA non-silencing control was transfected in pBabe–puro and HRAS V12 stable cell lines using lipofectamine 2000 transfection reagent according to the manufacturer's instructions (Invitrogen). Plasmin activity and invasion assays were performed 48 hours after transfection as described above. Statistical analysis was performed using two-way ANOVA with Tukey test of significance.

### Statistical analysis

Statistical significance was determined by Student's *t* test or two-way ANOVA. Results were considered significant if 2-tailed *P* values were less than 0.05. All data are expressed as mean ± StdDev.

## SUPPLEMENTARY MATERIALS


