# Supplementary material for: Cell surface protease activation during RAS transformation: Critical role of the plasminogen receptor, S100A10
Source: Oncotarget. 2016 Jun 24;7(30):47720–37. doi: 10.18632/oncotarget.10279 (PMC5216974; doi:10.18632/oncotarget.10279)
Supplement: Supplementary file 1 [file oncotarget-07-47720-s001.pdf]

Cell surface protease activation during RAS transformation: Critical role of the plasminogen receptor, S100A10

Supplementary Materials

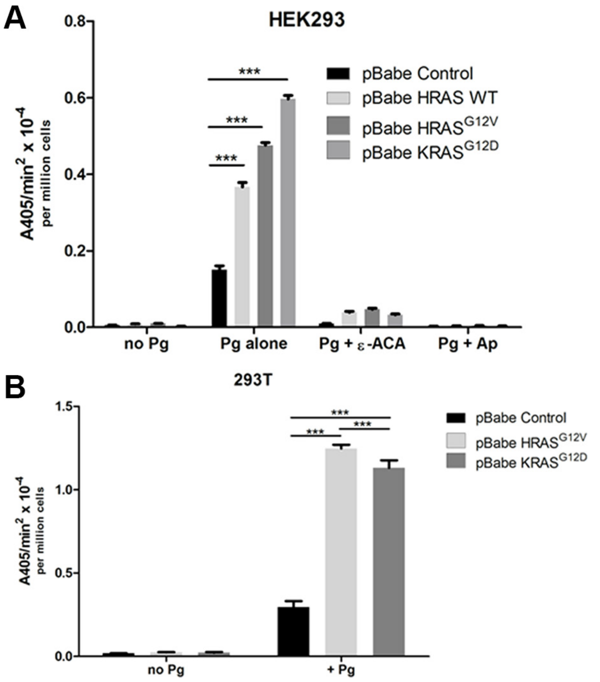

Supplementary Figure S1: Plasmin generation at the cell surface of oncogenic RAS expressing cells. Plasmin generation assay by HEK 293 (Figure 2A) or 293T (Figure 2B) cells transduced with empty vector retrovirus (pBabe control), wild type HRAS (HRAS WT), oncogenic HRAS GV12 expressing retrovirus (HRAS^{G12V}) and oncogenic KRAS G12D (KRAS^{G12D}) and stably selected with puromycin. Cells were either plated in 96 well plates for two days (HEK 293) before assay or released with enzyme free cell dissociation buffer and plated in 96 well plates in suspension for the assay (293T). Cells were either mock treated or treated with aprotinin (Ap) (2.2 µM), ϵ-ACA (100 mM) as indicated. The cells were washed 3 times with incubation buffer (Hanks balanced salt solution containing 3 mM $CaCl_2$ and 1 mM $MgCl_2$) and incubated with 0.5 µM glu-plasminogen for 20–30 minutes with or without aprotinin and ϵ-ACA before the addition of 500 µM plasmin substrate S2251 (Chromogenix, Diapharma Group). The rate of plasmin generation was determined from the slope of the A405nm vs time progress curve ($N = 4$). Statistical analysis was performed by two-way ANOVA.

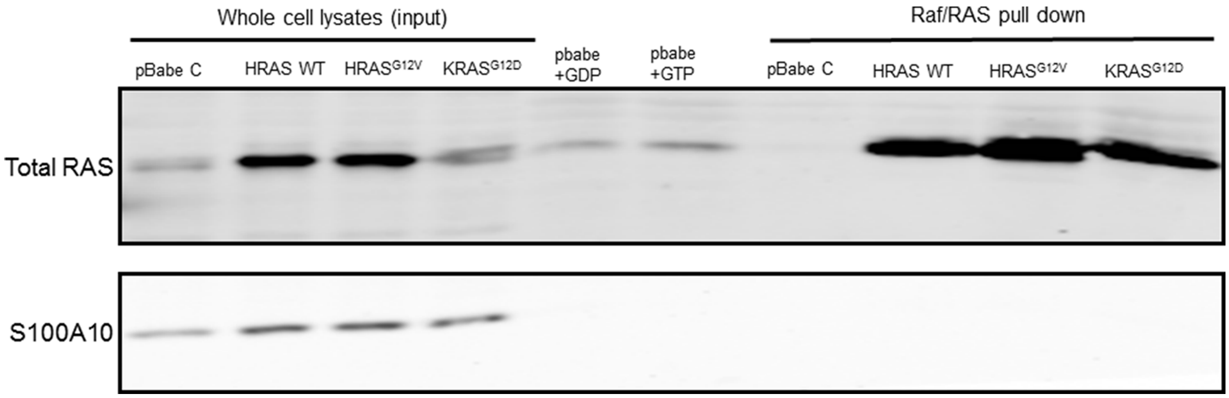

Supplementary Figure S2: RAS activity in RAS expressing HEK 293 cells. HEK 293 cells stably expressing oncogenic HRAS G12V (HRAS^{G12V}) and oncogenic KRAS G12D (KRAS^{G12D}) and empty vector (pBabe control) were plated, lysed and Ras/Raf pull down was carried as per the manufacturer's instructions (Millipore). GTP and GDP loaded empty vector cell lysates were used as positive and negative control for the pull down.

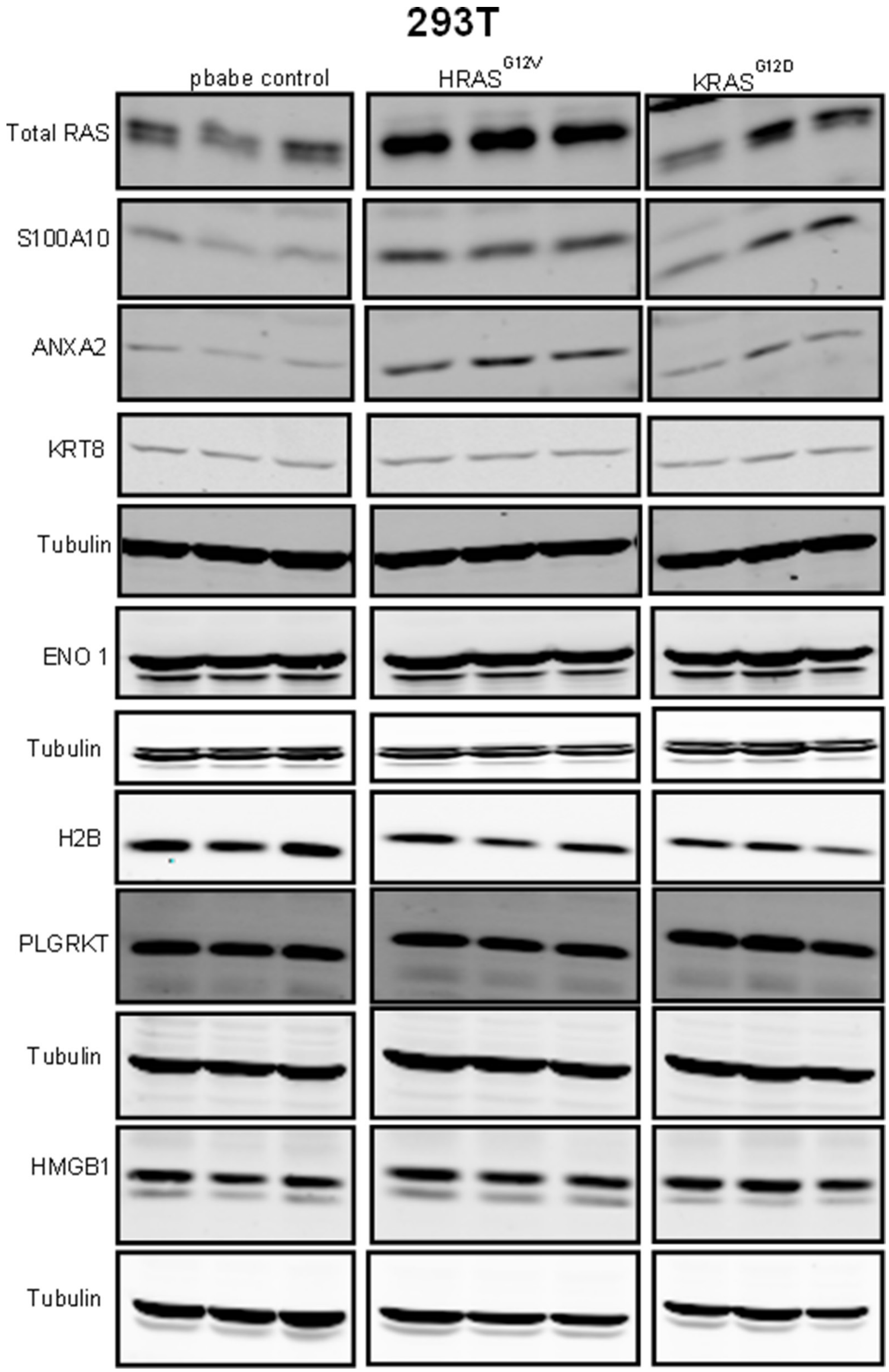

Supplementary Figure S3: Regulation of plasminogen receptor expression by RAS in 293T cells. 293T cells expressing empty vector (pBabe control), wild type HRAS (HRAS WT), oncogenic HRAS G12V (HRASG12V) and oncogenic KRAS G12D ($KRAS^{G12D}$) were plated, and after three days, lysed and subjected to Western blotting. The membrane was probed with primary antibodies as indicated and subsequently probed with IR-dye conjugated secondary antibodies scanned using LI-COR Odyssey scanner. Results are presented in triplicate.

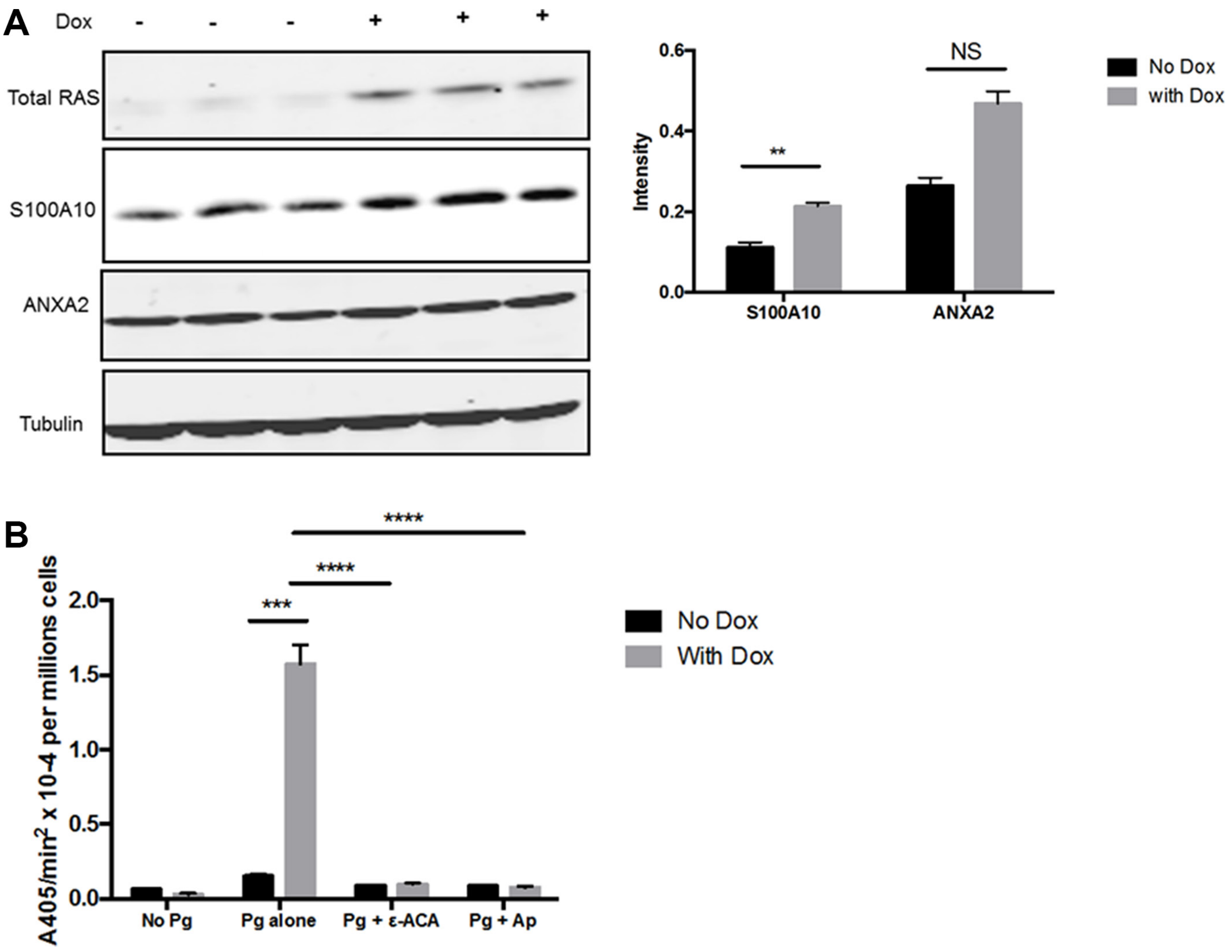

Supplementary Figure S4: Induction of p11 by oncogenic KRAS (KRAS^{G12D}) in pancreatic cancer cells. iKRAS murine cells (AK 196) were a kind gift from Dr. Alex Kimmelman (Division of Genomic Stability and DNA repair, Department of Radiation Oncology, Dana-Farber Cancer Institute, Boston, MA). The cells were seeded at 1×10^4 cells per well of a 6-well plate for experiments requiring cell lysates and 1×10^3 cells per well of a 96 well plate for plasmin generation assay. The cells were induced with 2 µg/ml of doxycycline while plating and were induced every subsequent day. Four days after induction, the cells were lysed in lysis buffer and protein content determined using the Bradford assay. (**A**) The total protein (20–30 ug) was separated using 10–20% gradient gel, followed by transfer to a nitrocellulose membrane. The membrane was probed with primary antibodies as indicated and subsequently probed with IR-dye conjugated secondary antibodies and scanned using Li-COR Odyssey imager. The band intensities were quantified using the software from Li-COR. (**B**) For plasmin generation assay, cells were either mock treated or treated with aprotinin (Ap) (2.2 µM), ϵ-ACA (100 mM) as indicated. The cells were washed 3 times with incubation buffer (Hanks balanced salt solution containing 3 mM $CaCl_2$ and 1 mM $MgCl_2$) and incubated with 0.5 µM glu-plasminogen for 20–30 minutes with or without aprotinin and ϵ-ACA followed by the addition of 500 µM plasmin substrate S2251 (Chromogenix, Diapharma Group). The rate of plasmin generation was determined from the slope of the A_{405} nm vs time^2 progress curve ($N = 4$). Statistical analysis was performed by Student t-test.

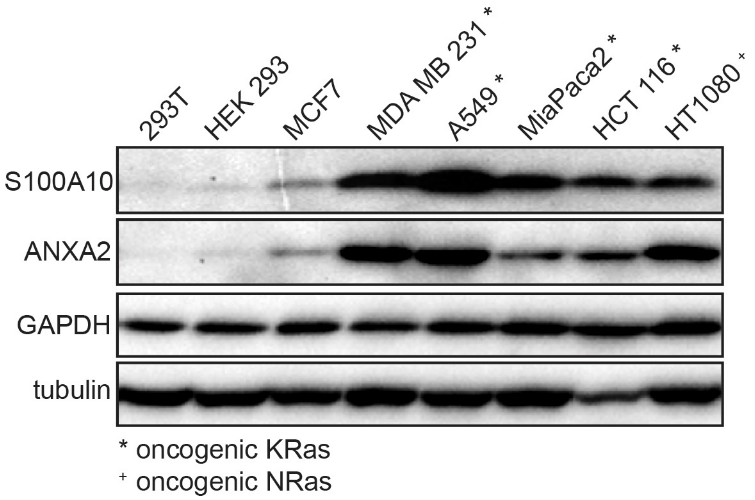

Supplementary Figure S5: S100A10 levels in human cancer cell lines. Protein lysates of a panel of well-established human cancer cell lines were prepared as described in materials and methods. 20 µg of each protein lysate was subjected to SDS-PAGE, transferred onto a nitrocellulose membrane and analyzed by western blotting with the antibodies indicated.

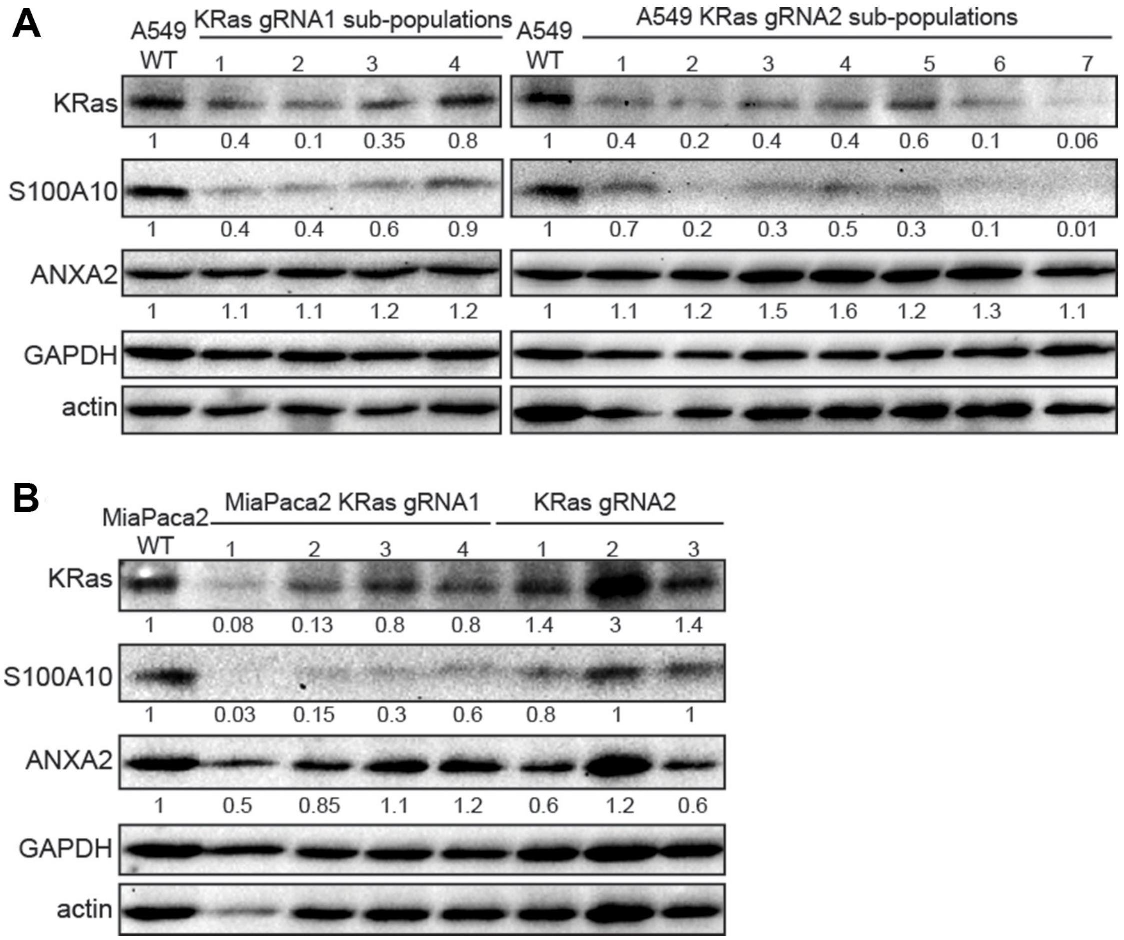

Supplementary Figure S6: Effect of KRAS knockdown on S100A10 levels. (**A**) A549 lung cancer cells or (**B**) MiaPaca2 pancreatic cancer cells were transfected with pKRAS gRNA1-px459 V2 (KRas gRNA1) or pKRAS gRNA2-px459 V2 (KRas gRNA2) as described in materials and methods. Cells were selected with 5 µg/ ml puromycin and serial dilutions were performed (sub-populations). Protein lysates of the different pools of cells (sub-populations) were prepared and 20 µg of each protein lysate or of each wild-type cell line were subjected to SDS-PAGE, transferred onto a nitrocellulose membrane and analyzed by western blotting with the antibodies indicated. Quantification of protein bands was performed using Image J software.

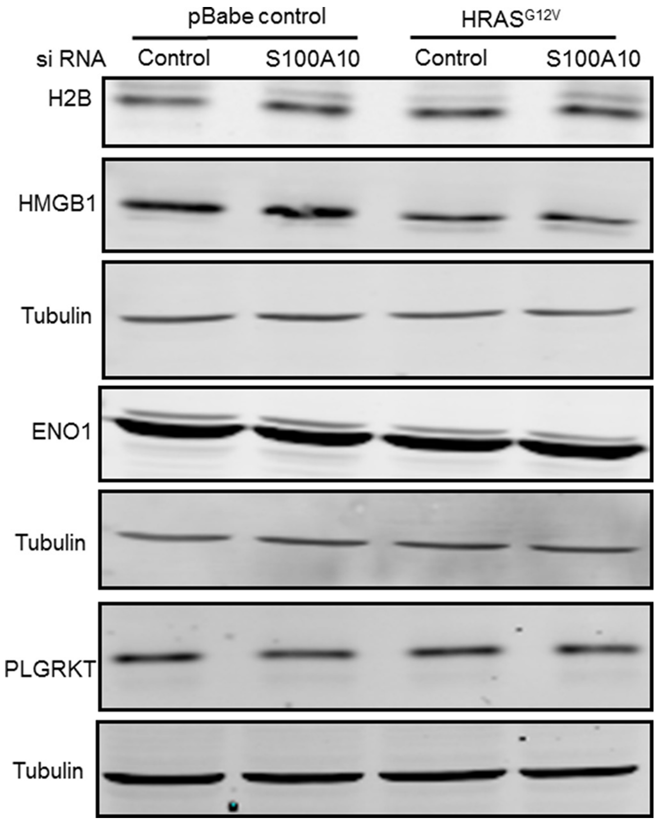

Supplementary Figure S7: Plasminogen receptor expression after knockdown of S100A10. 293T cells stably expressing oncogenic HRAS G12V (HRAS^{G12V}) and empty vector (pBabe control) were transfected with 4 μM of pre-designed siRNA (Ambion) specific for S100A10 (S100A10 siRNA) and a non-silencing siRNA control (siRNA control) using Lipofectamine 2000 transfection reagent as per manufacturer's instructions (Invitrogen). Cell lysates were prepared 48 hours after transfection, and total levels of non-neuronal enolase (ENO1), histone H2B, HMGB-1 were detected by Western blotting.
